# Recent 5‑year trends in biliary tract cancer survival rates: An analytical big data survey

**DOI:** 10.3892/mi.2025.214

**Published:** 2025-01-10

**Authors:** Ji Yoon Lee, Ju Won Kim

**Affiliations:** 1 Department of Biostatistics, Korea University College of Medicine, Seoul 02841, Republic of Korea; 2 Division of Oncology/Hematology, Department of Internal Medicine, Korea University Anam Hospital, Korea University College of Medicine, Seoul 02841, Republic of Korea

**Keywords:** biliary tract cancer, cholangiocarcinoma, big data, survival, risk factor

## Abstract

Biliary tract cancer (BTC), also known as cholangiocarcinoma, is a relatively rare type of cancer with a poor prognosis. Despite the combination of chemotherapy and advances in targeted therapy, which have potentially improved the prognosis of patients with BTC, research on outcomes remains inadequate. The present study thus analyzed the survival trends of patients with BTC. The present study used anonymized data from a public national database and focused on 13,600 individuals diagnosed with BTC between 2015 and 2020. The overall and 1-year mortality rates were analyzed according to cancer anatomic sites, along with the impact of comorbidities, such as diabetes and hepatitis on these rates. A total of 13,600 patients were included in the analysis; 26.31% of the patients had intrahepatic BTC, 27.46% had extrahepatic BTC and 46.24% had gallbladder (GB) cancer. For all BTC types, the 1-year survival hazard ratio (HR) in 2018 was 0.992 compared with that in 2015, and 0.986 in 2019. Compared with intrahepatic BTC, the 1-year survival rate was 0.349 for GB cancer and 0.641 for extrahepatic BTC. Patients with diabetes had an HR of 1.318 compared with those without diabetes. For patients with BTC previously diagnosed with GB stones, the survival HR was 0.902, compared to those without GB stones. On the whole, the analysis of national healthcare big data indicated an improvement in the overall prognosis of patients with BTC from 2018. Moreover, these data highlight that the prognosis of patients with BTC is influenced by the anatomical location of the cancer, and that co-existing medical conditions in patients affect the survival rate.

## Introduction

Biliary tract cancer (BTC), also known as cholangiocarcinoma, is a cancer of the digestive system that originates in the biliary tract, which transports bile from the liver to the digestive tract (1). BTC is a relatively rare type of cancer worldwide. However, high incidence rates have been reported in Asia, thus rendering it the sixth most common cause of cancer-related mortality in Korea (2).

BTC is also well known for its poor prognosis and the absence of an effective therapeutic target. Despite the benefits of targeted therapies observed in the majority of solid cancers, including lung, breast and even colorectal cancers, since the 2000s, well-known targeted therapies, such as anti-human epidermal growth factor receptor 2 (HER2) or anti-EGFR agents have only marginally benefited patients with BTC (3). While the age standardized mortality rate for lung cancer and colorectal cancer significantly decreased over the past decade, from 36.6 to 14.9 per 100,000 and from 10.1 to 7.2, respectively, the mortality rate of patients with BTC has exhibited only a minimal improvement (4.5 to 4.0 per 100,000, from 2010 to 2020) (2,4).

Due to its low incidence rate and poor prognosis, BTC has been overlooked in major clinical studies. Limited research and a lack of the accurate understanding of its current epidemiological characteristics are also key issues with respect to BTC treatment. With various chemotherapy combinations and the discovery of therapeutic targets through next-generation sequencing, along with the use of immune checkpoint inhibitors, the treatment landscape for biliary cancer is evolving (5). However, there is a paucity of in-depth studies in this area. Although reports indicate that PD-L1 expression and immune infiltration influence the prognosis of patients with liver cancer including BTC (6-8), conclusive results require further validation through prospective clinical trials and translational studies.

Korea, along with Thailand and Chile, has one of the highest incidences of BTC worldwide (14.5 per 100,000) (1). These regions exhibit differences in etiology and genomic landscape, which in turn affect the overall prognosis of patients with BTC. Therefore, regional exploration is essential to understand these variations (9,10). The National Health Information Database (NHID) of the National Health Insurance Service (NHIS) also contributes to the positioning of Korea as a valuable cohort for BTC research. The present study aimed to investigate the recent trends in the prognosis of patients with BTC and explores the differences based on patient comorbidities and tumor anatomic sites.

## Patients and methods

### Data source

The data used in the present study were obtained from the NHIS of Korea. Korean citizens are obligated to enroll in the NHIS for healthcare services, which cover ~97% of the population of Korea. NHIS data include information from all hospitals, including inpatient and outpatient records, using the International Statistical Classification of Diseases and Related Health Problems, Tenth Revision (ICD-10) codes.

The NHIS database contains various types of information, such as diagnosis, hospitalization and outpatient treatments, medical expenses, prescribed medications, performed surgeries, and patient demographics such as sex and age, as well as the date of death when applicable. This information is stored based on the content billed by the medical institutions. The database was provided to researchers with all personal information, such as names, resident registration numbers, addresses and phone numbers anonymized or removed.

The study protocol was approved by the Institutional Review Board of Korea University Anam Hospital (Seoul, Korea; IRB no. 2021AN0431).

### Study population

The present study included patients diagnosed with BTC between 2015 and 2020. BTC was defined as one of the following primary diagnostic ICD-10 codes: C22.1 (intrahepatic BTC), C23.X [gallbladder (GB) cancer], or C24.X (extrahepatic BTC). The date of claim registration in the NHIS database was assumed to be the date of diagnosis. Data from 2004 to 2014 were considered a washout period to account for the absence of new drugs and to obtain diagnostic data. Patients were included if they were aged ≥20 years at the time of their first diagnosis of BTC and underwent surgery within 1 year before or after the diagnosis, or were administered gemcitabine hydrochloride or cisplatin following the diagnosis (n=38,259). Among these, 24,659 patients who developed other types of cancer following BTC were excluded, thus leaving a final cohort of 13,600 patients for analysis.

Demographic variables, including age and sex were also assessed. Previous medical history included diabetes, hepatitis and gallstones. In the case that the corresponding disease codes existed prior to the diagnosis of cholangiocarcinoma, they were considered part of the medical history.

The primary outcome variables were overall and 1-year mortality rates. The analyses included comparisons between individuals who only underwent surgery and those who only received medication, comparisons based on the year of diagnosis, comparisons based on diagnosis codes, and analysis of mortality rates based on the presence of diabetes, hepatitis, and gallstones.

In the case of individuals diagnosed with BTC, those who underwent surgical treatment were defined with the code ‘Q7380, Q7410, Q7342, Q7221, Q7222, Q7223, Q7224 and Q7225’. Among those diagnosed with BTC, medications were defined using the medication codes for ‘gemcitabine (164901BIJ, 164902BIJ, 164903BIJ, 164904BIJ, 164930BIJ, 164931BIJ and 164932BIJ)’ and ‘cisplatin (134501BIJ, 134502BIJ, 134503BIJ, 134504BIJ, 134505BIJ, 134530BIJ, 134531BIJ, 134532BIJ, 134533BIJ and 134534BIJ)’. For diabetes, the codes R81.X, E10.X and E11.X were used; chronic hepatitis B virus (HBV) was identified using B18.X; gallstones were identified using K80.X.

### Statistical analysis

Continuous variables are presented as the mean ± standard deviation, and categorical variables are presented as frequencies and percentages. Cox proportional hazard regression models were used to compare the risk of disease outcomes between the groups, which allowed for the calculation of hazard ratios (HRs) and 95% confidence intervals (CIs). Before analyzing the Cox model, a log-rank test was used on the Kaplan-Meier survival curves to confirm differences among groups, where all groups exhibited a P-value <0.05, thus satisfying the proportional hazards assumption. This analysis was performed as an unadjusted analysis without any adjustments. The reason for not using an adjusted analysis is that the present study primarily focused on identifying raw correlations. All statistical analyses were performed using the SAS software (version 9.4; SAS Institute). All P-values were two-sided, and a P-value <0.05 was considered to indicate a statistically significant difference.

## Results

### Study population

Data on patients who had been diagnosed with BTC between 2015 and 2019 were collected from the NHID. Patients diagnosed with other types of cancer were excluded from data collection, resulting in a final cohort of 10,222 patients for analysis. Among these patients, 2,614 patients (25.57%) had intrahepatic BTC, 2,757 patients (26.97%) had extrahepatic BTC and 4,851 patients (47.46%) had GB cancer. The clinical profiles of the final cohort are summarized in Table I.

The mean age of cohort was 66.94 (SD 11.58) years, and 54.94% were male. Prior to being diagnosed with BTC, 67.01% of the patients had diabetes mellitus, 26.38% had hepatitis, and 55.45% had GB stones.

### Survival probability according to the year of diagnosis

**Figure 1:**
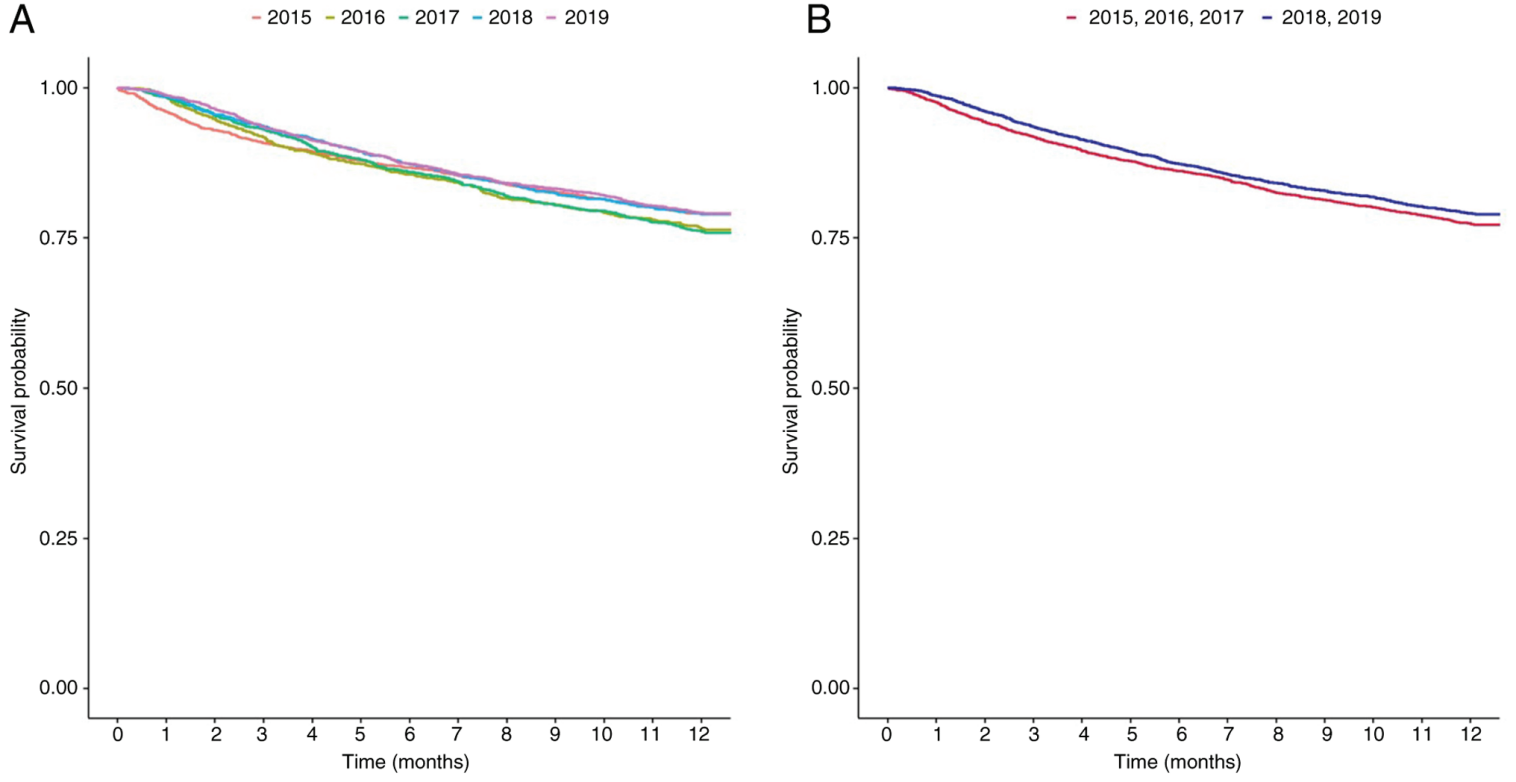
Yearly differences in the clinical outcomes of patients with biliary tract cancer. (A) Group by yearly differences in clinical outcomes. (B) Kaplan-Meier curves of unadjusted 1-year mortality.

The HR of the 1-year survival rates of the patients diagnosed each year are presented in Fig. 1 and Tables SI and SII: 1.133 (95% CI, 0.988-1.299) for 2016, 1.15 (95% CI, 1.008-1.311) for 2017, 0.992 (95% CI, 0.869-1.131) for 2018, and 0.986 (95% CI, 0.868-1.119) for 2019, with 2015 set as the reference year. Numerically, the 1-year survival rate of the patients with BTC increased from 20.95% in 2015 to 24.11% in 2017, and then decreased to 21.07% in 2018, as shown in Fig. 1A and Table SI. The HR of the 1-year survival rate of the relatively recently diagnosed patients was lower than that of the patients diagnosed earlier, and the difference was statistically significant (HR, 0.908; 95% CI, 0.836-0.987; P=0.0237) (Fig. 1B and Table SII). Table SII presents the P-value derived from the univariate log-rank test, whereas the P-value reported in the main text corresponds to that obtained from the Cox proportional hazards model.

### Survival probability according to treatment setting

In the analysis of the treatment setting, to compare surgery and chemotherapy, 491 patients who received no treatment and 856 patients who underwent both surgery and chemotherapy were excluded, resulting in a total of 10,222 patients being included in the analysis. When the patients were categorized according to the treatment they received for BTC, 9,451 patients received surgery only, and 2,802 received chemotherapy only (Table II). The survival probability was markedly lower in the chemotherapy-only group than in the surgery-only group (HR, 4.857; 95% CI, 4.508-5234; P<0.001, Fig. S1).

### Survival probability according to anatomic site

**Figure 2:**
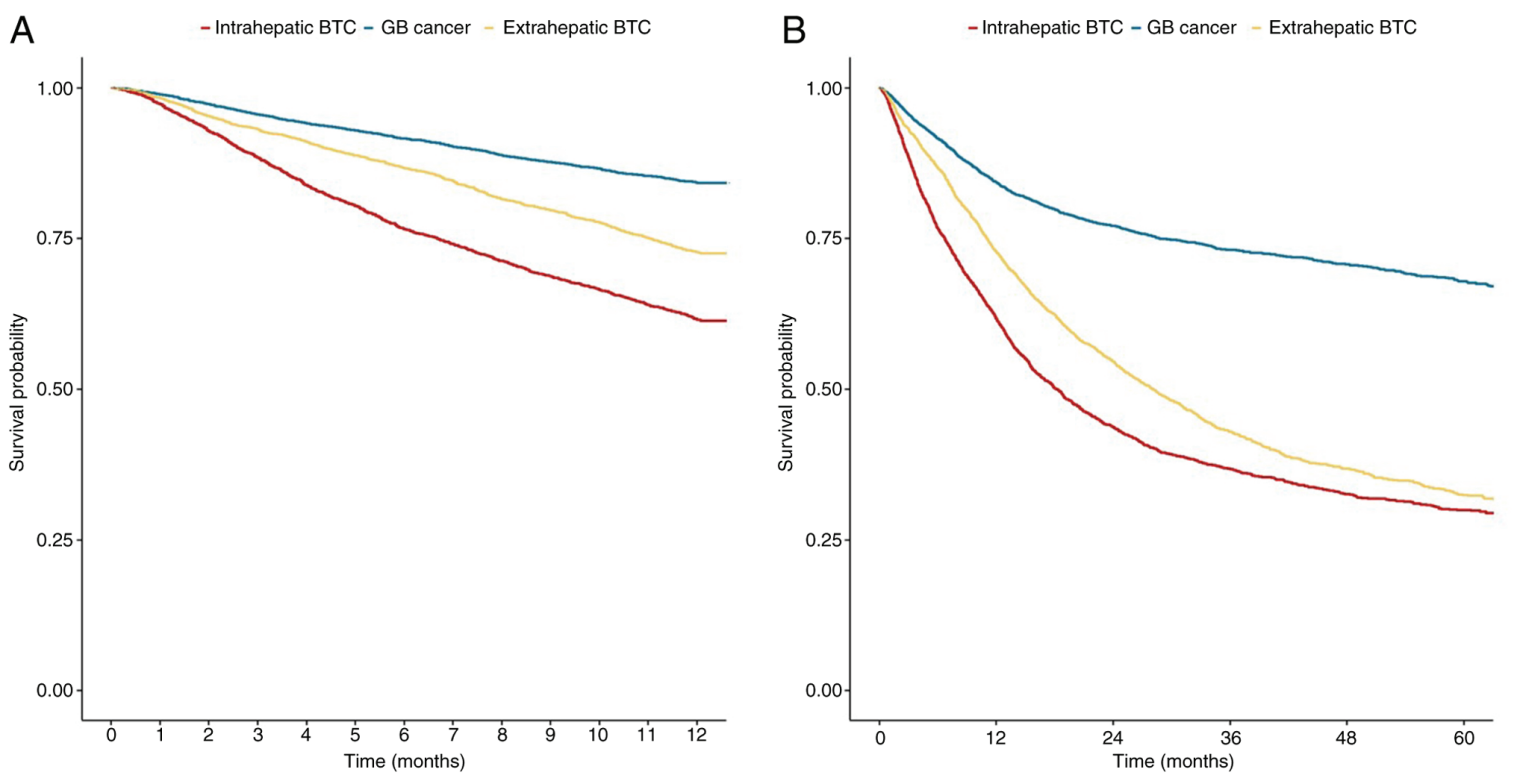
Anatomic site differences in the clinical outcomes of patients with BTC. Kaplan-Meier curves of unadjusted (A) 1-year, and (B) overall mortality. BTC, biliary tract cancer; GB, gallbladder.

The survival rates of the patients categorized according to anatomical tumor sites are presented in Fig. 2. The data for intrahepatic BTC, extrahepatic BTC and GB cancer are also shown in (Tables SIII and SIV). The 1-year survival probability was the lowest in GB cancer (HR, 0.349; 95% CI, 0.32-0.381; P<0.001), followed by extrahepatic BTC (HR, 0.641; 95% CI, 0.589-0.698; P<0.001) when intrahepatic BTC was used as a reference (Fig. 2A and Table SIII). The median survival probability was not reached for intrahepatic BTC (Fig. 2B).

### Survival probability according to comorbidities

**Figure 3:**
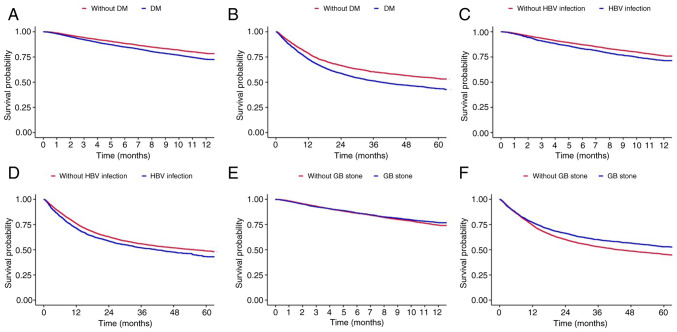
Survival probability of 1-year mortality and overall mortality, group by (A and B) diabetes mellitus, (C and D) HBV infection, and (E and F) GB stones. DM, diabetes mellitus; HBV, hepatitis B virus infection; GB, gallbladder.

The present study conducted a subgroup analysis according to the comorbidities of the patients with BTC (Fig. 3 and Table SV, Table SVI, Table SVII, Table SVIII, Table SIX and Table SX). Patients who had been diagnosed with diabetes mellitus prior to being diagnosed with BTC had a poorer prognosis than those who had not (HR, 1.318; 95% CI, 1.225-1.418; P<0.001; Fig. 3A and Table SV). The analysis of overall survival revealed the same tendency (HR, 1.322; 95% CI, 1.252-1.397; P<0.001; Fig. 3B and Table SVI). The median overall survival of the patients without diabetes mellitus was 76.587 months (95% CI, 68.12-85.85), while that of the patients with diabetes mellitus was 38.889 months (95% CI, 35.88-41.87) (Fig. 3B).

Patients who had chronic HBV infection also exhibited a higher HR than patients who did not have HBV infection (HR, 1.249; 95% CI, 1.147-1.36; P<0.001; Fig. 3C and Table SVII). The median overall survival of the patients who had known HBV infection was 55.556 months (95% CI, 50.73-40.71; Fig. 3D). Patients with known GB stones prior to being diagnosed with BTC had a decreased risk of mortality (HR, 0.902; 95% CI, 0.834-0.976; P=0.01; Fig. 3E and F, and Table SIX).

## Discussion

The present study examined the 1-year survival of patients diagnosed with BTC over a period of 5 years, beginning from 2015. The present study investigated the impact of various factors on the survival rates of patients with BTC. Consistent yearly survival rates were observed with a slight decline after 2018. This decline was statistically significant when comparing the pre- and post-2018 data. Intrahepatic BTC was associated with the shortest survival time, whereas a history of diabetes or chronic HBV infection negatively affected survival. However, cholelithiasis was associated with an improved survival.

BTC is well known for its poor prognosis, with patients with stage 4 disease having a 5-year survival rate <10%.2 Several clinical characteristics contribute to this poor prognosis: Diagnosis often occurs at an advanced stage due to the absence of early symptoms, the heterogeneous nature of cancer with a wide range of anatomies (from intrahepatic to the ampulla of Vater) (1), and the limitation of cytotoxic chemotherapy due to the lack of effective therapeutic targets. While advancements in diagnostic techniques, active health screening and the widespread adoption of genetic analyses, such as next-generation sequencing have addressed numerous challenges regarding BTC, the extent of the improvement in the prognosis of patients with BTC remains elusive.

**Figure 4:**
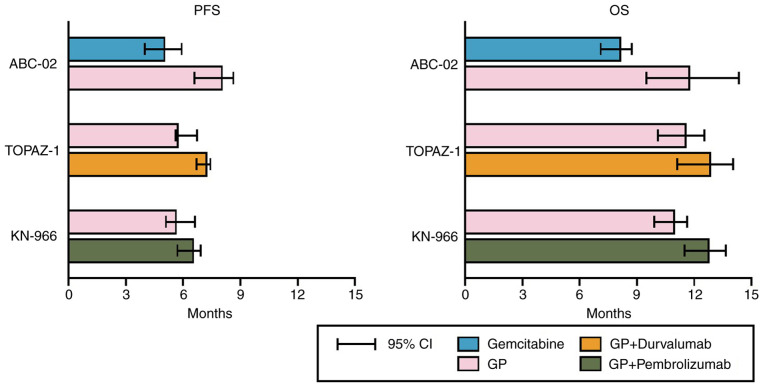
Results of the key phase 3 palliative chemotherapy clinical trial for biliary tract cancer (11,13,14). PFS, progression-free survival; OS, overall survival; GP, gemcitabine and cisplatin.

From a therapeutic perspective, combination therapy using gemcitabine and cisplatin (GP) has been pivotal since 2010. This combination has been shown to lead to improved response rates and survival benefits over gemcitabine monotherapy (medial overall survival, 11.7 vs. 8.1 months; HR, 0.64; P<0.001), establishing a standard palliative treatment approach (11). Since then, for over a decade, no treatment strategy has surpassed this combination therapy. A few additional benefits of epidermal growth factor receptor and vascular endothelial growth factor receptor inhibitors have been observed (12). It was not until 2022 that the addition of durvalumab to GP was proven to extend survival (medial overall survival, 12.8 months) (13); subsequently, the combination with pembrolizumab also began to yield promising results (medial overall survival, 12.7 months, Fig. 4) (14).

The absence of effective subsequent therapy following the initial treatment also contributes to the poor prognosis of patients with BTC. Based on a phase 2 clinical trial published in 1998, fluorouracil (5-FU)-based regimens have been widely used empirically (15); however, owing to the rarity of the affected population, obtaining well-designed phase 3 data has been challenging. Since 2014, the ABC-06 trial has tested the FOLFOX regimen (5-FU, leucovorin and oxaliplatin), eventually yielding the first positive data for a second line therapeutic option in 2021(16).

However, in the era of precision oncology, BTC treatments are evolving. A number of basic studies have identified molecular targets that are potentially useful for target-directed therapies, such as the fibroblast growth factor receptor (*FGFR*), HER2, metabolic regulators, such as isocitrate dehydrogenase 1 and 2 (*IDH1/2*), and phosphatidylinositol-4,5-bisphosphate 3-kinase catalytic subunit alpha (17-21). Among these, *FGFR2*-targeting agents (e.g., pemigatinib and futibatinib) (22-24) and IDH1-targeting agents (e.g., ivosidenib) have been approved by the FDA (25,26). HER2 overexpression is also a major treatment target in GB cancer (27), and not only existing HER2-targeted agents (e.g., trastuzumab and pertuzumab) (28,29), but also novel agents (e.g., zanidatamab) are being used for treatment (30). Ethnic differences in therapeutic targets have been consistently reported (31), and particularly in Koreans, the prevalence of IDH1/2 and FGFR2 aberrations is lower than that typically reported (9). However, with the widespread use of next-generation sequencing (NGS), efforts to identify patients who could benefit from targeted therapy are continuing, and the impact of these therapies on prognosis is a subject that requires thorough investigation. Owing to these efforts, the survival rates of patients with BTC are steadily increasing, albeit at a gradual pace. Annual cancer statistics in Korea have also reported that the 5-year relative survival rate of patients with BTC exhibited an upward trend until the period of 2011-2015 (29.1%) (32); however, from to 2015-2019 (28.5%), it exhibited a decreasing tendency (-0.6%) (33). Based on our data, it is plausible that these changes originated in 2018. Novel drugs, such as nanoliposomal irinotecan (NIFTY) (34) and immune checkpoint inhibitor clinical trials (TOPAZ-1 and KEYNOTE-966) (13,14), have recruited patients in Korea since 2018. TOPAZ-1 and KEYNOTE-966 demonstrated that a combination of immune checkpoint inhibitors and chemotherapy significantly extended the survival rates of patients with advanced BTC (13,14). Continuous real-world follow-up studies are required to determine the changes in the survival rates of patients with BTC following immunotherapy in clinical settings.

Anatomical heterogeneity has long been a challenging issue in BTC, with each cancer subtype exhibiting variable survival rates. In the present study, the patients with intrahepatic cholangiocarcinoma had the worst prognosis. This may be attributed to its tendency to present with fewer symptoms, such as jaundice or abdominal pain, leading to a delayed diagnosis. This finding aligns with the research results from the study by Tawarungruang *et al* (35), that tracked prognosis following surgical treatment (median survival time 21.8 months in distal cholangiocarcinoma vs. 12.4 months in intrahepatic cholangiocarcinoma). Anatomical heterogeneity is closely associated with molecular heterogeneity (36). However, the difference in the response to chemotherapy among anatomical subgroups in the ABC-02 and TOPAZ-1 trials was not distinct (11,13), indicating the need for further research in this area.

The subgroup analysis in the present study provided several insights. Diabetes is a well-known risk factor of BTC (37,38). In the present study, patients with pre-diagnosed diabetes exhibited a significantly reduced overall survival compared to those without diabetes (38.889 vs. 76.587 months). This suggests that diabetes not only functions as a risk factor for the development of BTC, but also significantly affects overall survival. Additionally, it was observed that patients with a history of cholelithiasis had an extended survival compared with those without. Regular follow-up sessions for known GB stones and the early detection of cancer due to symptoms, leading to prompt treatment, are considered to have contributed to the prolonged survival of these patients. However, the present study identified data-driven phenomena, not biological mechanisms, necessitating cautious interpretation. Further *in vivo* and prospective studies are required to validate these findings.

The present study had several limitations, which should be mentioned. The present study was unable to obtain information on the clinical or pathological stages of cancer, which significantly affect survival and prognosis. The authors attempted to overcome this limitation through precise operational definitions by using various treatments. NHIS research utilizes data based on insurance claims, which means that data for items not covered by insurance are not provided. Additionally, sensitive data that could identify patients were not disclosed to protect personal information, such as family history or socioeconomic status. Detailed medical information specific to particular test results or specific medical conditions was unavailable for research purposes. Nevertheless, the lack of information was not concentrated in any one subgroup, but was applied to all subjects, which the authors believe lends value to the results as a gross outcome of macroscopic trends from big data.

In conclusion, the present study tracked the survival trends of patients with BTC using national healthcare big data, suggesting an improvement in the overall prognosis of patients with BTC since 2018. Additionally, it was revealed that the prognosis of patients with BTC varies depending on the anatomic site of the cancer, as well as the presence of underlying diseases, despite the absence of detailed pathologic information.

## Supplementary Material

Survival probability of patients treated with chemotherapy only and surgery only.

One-year survival rates of the patients (Fig. 1A).

One-year survival rates of the patients (Fig. 1B).

One-year survival rates of the patients (Fig. 2A).

Overall survival rates of the patients (Fig. 2B).

One-year survival rates of the patients (Fig. 3A).

Overall survival rates of the patients (Fig. 3B).

One-year survival rates of the patients (Fig. 3C).

Overall survival rates of the patients (Fig. 3D).

One-year survival rates of the patients (Fig. 3E).

Overall survival rates of the patients (Fig. 3F).

## Figures and Tables

**Table I:** Clinical characteristics and comorbidities of the patients with BTC.

Characteristics	Patients (n=10,222)
Age, years; mean (SD)	66.94 (11.58)
Sex	
Male	5,616 (54.94)
Female	4,606 (45.06)
Incidence year	
2015	2,091 (20.46)
2016	1,621 (15.86)
2017	1,875 (18.34)
2018	2,131 (20.85)
2019	2,504 (24.50)
Medical history	
Diabetes mellitus	6,850 (67.01)
Chronic hepatitis B virus infection	2,696 (26.38)
Cholelithiasis	5,668 (55.45)
Diagnosis of BTC	
Intrahepatic cholangiocarcinoma	2,614 (25.57)
Gallbladder cancer	4,851 (47.46)
Extrahepatic cholangiocarcinoma	2,757 (26.97)

BTC, biliary tract cancer.

**Table II:** One-year survival rates of the patients.

Treatment	No. of events/n (%)	Log rank z-test statistic	Log rank test P-value	HR (95% CI)
Surgery only	1,133/9,451 (11.99%)	45.8484	<0.0001	Ref.
Chemotherapy only	1,485/2,802 (53.00%)			4.857 (4.508-5.234)

HR, hazard ratio; CI, confidence interval.

## Data Availability

The data generated in the present study may be requested from the corresponding author.
