# Supplementary figures and images for: Recent 5‑year trends in biliary tract cancer survival rates: An analytical big data survey
Source: Med Int (Lond). 2025 Jan 10;5(2):15. doi: 10.3892/mi.2025.214 (PMC11775868; doi:10.3892/mi.2025.214)

Figure S1. Survival probability of patients treated with chemotherapy only and surgery only.

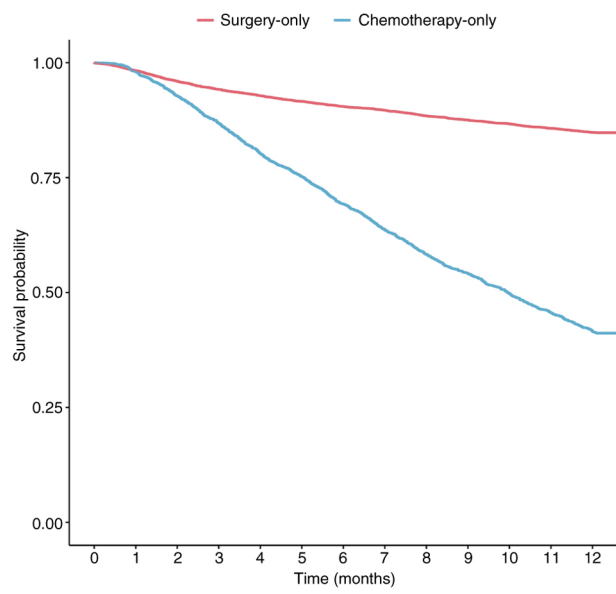

Supplement: Survival probability of patients treated with chemotherapy only and surgery only. [file Supplementary_Data1.pdf]
