# Supplementary material for: Recent 5‑year trends in biliary tract cancer survival rates: An analytical big data survey
Source: Med Int (Lond). 2025 Jan 10;5(2):15. doi: 10.3892/mi.2025.214 (PMC11775868; doi:10.3892/mi.2025.214)
Supplement: One-year survival rates of the patients (Fig. 1A). [file Supplementary_Data2.pdf]

Table SI. One-year survival rates of the patients (Fig. 1A).				
Year	No. of events/n (%)	Log rank z-test statistic	Log rank test P-value	HR (95% CI)
2015	438/2,091 (20.95%)	3.2450	0.0324	Ref.
2016	383/1,621 (23.63%)			1.133 (0.988-1.299)
2017	452/1,875 (24.11%)			1.15 (1.008-1.311)
2018	449/2,131 (21.07%)			0.992 (0.869-1.131)
2019	526/2,504 (21.01%)			0.986 (0.868-1.119)

HR, hazard ratio; CI, confidence interval.

Table SII. One-year survival rates of the patients (Fig. 1B).				
Year	No. of events/n (%)	Log rank z-test statistic	Log rank test P-value	HR (95% CI)
2015, 2016, 2017	1,273/5,587 (20.95%)	2.2747	0.0229	Ref.
2018, 2019	975/4,635 (21.01%)			0.908 (0.836-0.987)
HR, hazard ratio; CI, confidence interval.				

Table SIII. One-year survival rates of the patients (Fig. 2A).				
Type of BTC	No. of events/n (%)	Log rank z-test statistic	Log rank test P-value	HR (95% CI)
Intrahepatic BTC	1,250/3,578 (34.94%)	24.8910	<0.0001	Ref.
GB cancer	895/6,288 (14.23%)			0.349 (0.32-0.381)
Extrahepatic BTC	927/3,734 (24.83%)			0.641 (0.589-0.698)

BTC, biliary tract cancer; GB, gallbladder; HR, hazard ratio; CI, confidence interval.

Table SIV. Overall survival rates of the patients (Fig. 2B).

Type of BTC	No. of events/n (%)	Log rank z-test statistic	Log rank test P-value	HR (95% CI)
Intrahepatic BTC	2,036/3,578 (56.90%)	36.1557	<0.0001	Ref.
GB cancer	1,537/6,288 (24.44%)			0.321 (0.3-0.343)
Extrahepatic BTC	1,897/3,734 (50.80%)			0.787 (0.739-0.838)

BTC, biliary tract cancer; GB, gallbladder; HR, hazard ratio; CI, confidence interval.

Table SV. One-year survival rates of the patients (Fig. 3A).				
DM status	No. of events/n (%)	Log rank z-test statistic	Log rank test P-value	HR (95% CI)
Without DM	1,138/5,740 (19.83%)	7.4202	<0.0001	Ref.
With DM	1,934/7,860 (24.61%)			1.318 (1.225-1.418)
DM, diabetes mellitus; HR, hazard ratio; CI, confidence interval.				

Table SVI. Overall survival rates of the patients (Fig. 3B).

DM status	No. of events/n (%)	Log rank z-test statistic	Log rank test P-value	HR (95% CI)
Without DM	2,081/5,740 (36.25%)	10.0554	<0.0001	Ref.
With DM	3,389/7,860 (43.12%)			1.322 (1.252-1.397)

DM, diabetes mellitus; HR, hazard ratio; CI, confidence interval.

Table SVII. One-year survival rates of the patients (Fig. 3C).

HBV status	No. of events/n (%)	Log rank z-test statistic	Log rank test P-value	HR (95% CI)
Without HBV infection	2,386/1,0889 (21.91%)	5.1458	<0.0001	Ref.
With HBV infection	686/2,711 (25.30%)			1.249 (1.147-1.36)

HBV, hepatitis B virus, HR, hazard ratio; CI, confidence interval.

Table SVIII. Overall survival rates of the patients (Fig. 3D).				
HBV status	No. of events/n (%)	Log rank z-test statistic	Log rank test P-value	HR (95% CI)
Without HBV infection	4,345/1,0889 (39.90%)	4.2824	<0.0001	Ref.
With HBV infection	1,125/2,711 (41.50%)			1.154 (1.081-1.232)
HBV, hepatitis B virus, HR, hazard ratio; CI, confidence interval.				

Table SIX. One-year survival rates of the patients (Fig. 3E).				
GB stone status	No. of events/n (%)	Log rank z-test statistic	Log rank test P-value	HR (95% CI)
Without GB stones	2,197/9,428 (23.30%)	2.5772	0.01	Ref.
With GB stones	875/4,172 (20.97%)			0.902 (0.834-0.976)
GB, gallbladder; HR, hazard ratio; CI, confidence interval.				

Table SX. Overall survival rates of the patients (Fig. 3F).

GB stone status	No. of events/n (%)	Log rank z-test statistic	Log rank test P-value	HR (95% CI)
Without GB stones	3,935/9,428 (41.74%)	6.6615	<0.0001	Ref.
With GB stones	1,535/4,172 (36.79%)			0.818 (0.771-0.868)

GB, gallbladder; HR, hazard ratio; CI, confidence interval.
